# Impact of Alcohol and Coffee Intake on the Risk of Advanced Liver Fibrosis: A Longitudinal Analysis in HIV-HCV Coinfected Patients (ANRS CO-13 HEPAVIH Cohort)

**DOI:** 10.3390/nu10060705

**Published:** 2018-05-31

**Authors:** Issifou Yaya, Fabienne Marcellin, Marie Costa, Philippe Morlat, Camelia Protopopescu, Gilles Pialoux, Melina Erica Santos, Linda Wittkop, Laure Esterle, Anne Gervais, Philippe Sogni, Dominique Salmon-Ceron, Maria Patrizia Carrieri, the ANRS CO13-HEPAVIH Cohort Study Group

**Affiliations:** 1 Aix Marseille Université, INSERM, IRD, SESSTIM, Sciences Economiques & Sociales de la Santé & Traitement de l’Information Médicale, 27 Bd Jean Moulin, 13005 Marseille, France; fabienne.marcellin@inserm.fr (F.M.); marie.costa@inserm.fr (M.C.); camelia.protopopescu@inserm.fr (C.P.); maria-patrizia.carrieri@inserm.fr (M.P.C.); 2 ORS PACA, Observatoire Régional de la Santé Provence-Alpes-Côte d’Azur, 27 Bd Jean Moulin, 13005 Marseille, France; 3 Service de Médecine Interne, Hôpital Saint André, Centre Hospitalier Universitaire de Bordeaux, Université de Bordeaux, 1 Rue Jean Burguet, 33000 Bordeaux, France; philippe.morlat@chu-bordeaux.fr; 4 ISPED, Inserm, Bordeaux Population Health Research Center, Team MORPH3EUS, UMR 1219, CIC-EC 1401, Université Bordeaux, F-33000 Bordeaux, France; linda.wittkop@u-bordeaux.fr; 5 Département des Maladies Infectieuses, Hôpital Tenon, 4, rue de la Chine, 75020 Paris, France; gilles.pialoux@aphp.fr; 6 Ministério da Saúde, Secretaria de Vigilância em Saúde, Departamento de Vigilância, Prevenção e Controle das IST, do HIV/Aids e das Hepatites Virais, Brasília 70719-040, Brazil; melinabtu@gmail.com; 7 Faculdade de Ciências da Saúde, Programa de Pós-Graduação em Saúde Coletiva, Universidade de Brasília, Brasília 70910-900, Brazil; laure.esterle@u-bordeaux.fr; 8 CHU de Bordeaux, Pole de Sante Publique, Service d’Information Medicale, F-33000 Bordeaux, France; 9 Service des Maladies Infectieuses et Tropicales, AP-HP, Hôpital Bichat Claude Bernard, 46 rue Henri Huchard, 75018 Paris, France; anne.gervais@aphp.fr; 10 INSERM U-1223, Institut Pasteur, Service d’Hépatologie, Hôpital Cochin, Université Paris Descartes, 27 rue du Faubourg Saint-Jacques, 75014 Paris, France; philippe.sogni@aphp.fr; 11 Service Maladies Infectieuses et Tropicales, AP-HP, Hôpital Cochin, Université Paris Descartes, 27 rue du Faubourg-Saint-Jacques, 75014 Paris, France; dominique.salmon@aphp.fr

**Keywords:** HIV-HCV co-infection, liver fibrosis, coffee, alcohol consumption

## Abstract

Background: Coffee intake has been shown to modulate both the effect of ethanol on serum GGT activities in some alcohol consumers and the risk of alcoholic cirrhosis in some patients with chronic diseases. This study aimed to analyze the impact of coffee intake and alcohol consumption on advanced liver fibrosis (ALF) in HIV-HCV co-infected patients. Methods: ANRS CO13-HEPAVIH is a French, nationwide, multicenter cohort of HIV-HCV-co-infected patients. Sociodemographic, behavioral, and clinical data including alcohol and coffee consumption were prospectively collected using annual self-administered questionnaires during five years of follow-up. Mixed logistic regression models were performed, relating coffee intake and alcohol consumption to ALF. Results: 1019 patients were included. At the last available visit, 5.8% reported high-risk alcohol consumption, 27.4% reported high coffee intake and 14.5% had ALF. Compared with patients with low coffee intake and high-risk alcohol consumption, patients with low coffee intake and low-risk alcohol consumption had a lower risk of ALF (aOR (95% CI) 0.24 (0.12–0.50)). In addition, patients with high coffee intake had a lower risk of ALF than the reference group (0.14 (0.03–0.64) in high-risk alcohol drinkers and 0.11 (0.05–0.25) in low-risk alcohol drinkers). Conclusions: High coffee intake was associated with a low risk of liver fibrosis even in HIV-HCV co-infected patients with high-risk alcohol consumption.

## 1. Background

Chronic hepatitis C virus (HCV) infection in patients co-infected with HIV who receive antiretroviral treatment (ART) accelerates hepatic complications including chronic inflammatory lesions of the liver, steatosis, liver fibrosis progression, liver cirrhosis and hepatocellular carcinoma (HCC) [1,2]. In addition, excessive alcohol consumption, which is associated with reduced liver function and steatosis in the general population, can increase the severity of fibrosis in HIV-HCV co-infected individuals due to the strong dose-response relationship between alcohol and liver fibrosis progression [3,4,5]. Furthermore, chronic alcohol consumption increases the risk of developing HCC, through inflammation of hepatic cells and metabolic disorders [6].

The consumption of certain beverages, such as coffee and green tea, has been shown to have hepatoprotective effects [7,8]. Coffee is one of the most consumed drinks in the world, especially in high-resource settings [9]. Coffee contains large amounts of bioactive compounds, including caffeine, diterpenes, melanoidins, and antioxidants, such as chlorogenic acids [10]. Dietary intake of coffee has been shown to be associated with human health [11], in particular with lower risk of mortality [12], cancer [13] and cardiovascular disease (CVD) [14]. Epidemiological studies have found an association between high coffee intake (≥3 cups per day) and lower levels of liver enzymes, including aspartate aminotransferase (AST), alanine aminotransferase (ALT) and gamma-glutamyl transferase (GGT), which are markers of liver function [15,16,17]. In recent years, coffee intake has also been shown to modulate both the effect of ethanol on serum GGT activities in alcohol consumers and the risk of alcoholic cirrhosis in patients with chronic diseases [18]. In the context of HIV-HCV co-infection, high coffee intake has been found to have important benefits in terms of better adherence to treatment, less perceived toxicity [19,20], reduced levels of liver enzymes and lower risk of insulin resistance [15,17]. Several meta-analyses have also shown that coffee consumption is associated with a significant delay in the progression of liver fibrosis [21] and a reduced risk of HCC [22]. 

To our knowledge, no longitudinal study has ever analyzed the concomitant effects of coffee intake and alcohol consumption on liver fibrosis severity in HIV-HCV co-infected patients. This study aimed to analyze the impact of the interaction between high coffee intake and alcohol consumption on advanced liver fibrosis (ALF) among co-infected patients.

## 2. Materials and Methods

### 2.1. Study Design

This study is based on longitudinal data collected in the prospective, multicenter, observational ANRS CO13 HEPAVIH cohort, which recruited 1293 adult HIV-HCV co-infected individuals from 21 hospital centers throughout France between January 2006 and June 2014 [23].

Inclusion criteria in the cohort were as follows: being aged 18 years or more, HIV-1 infection and chronic HCV co-infection. Patients who had already cleared HCV, i.e., those who had a sustained virological response (SVR) to previous HCV treatment and those who had spontaneously cleared HCV, could also be included if eligible.

The study population included participants in the cohort with at least one measurement of alcohol consumption and coffee intake during the five first years of cohort follow-up. Patients with a history of liver transplant or clinical signs of decompensated liver cirrhosis at enrolment were excluded.

### 2.2. Data Collection

Throughout the follow-up, clinical/biological and socio-behavioral data were collected from medical records (clinical visits were scheduled annually, or every six months for cirrhotic patients) and annual self-administered questionnaires, respectively.

#### 2.2.1. Outcome: Advanced Liver Fibrosis (ALF)

For the evaluation of liver fibrosis, we used patient age and serum markers—including ALT, AST, and platelets count—to calculate the FIB-4 index [24]. ALF was defined at each visit as a FIB-4 index >3.25.

#### 2.2.2. Covariates

##### Clinical Variables

Clinical variables considered in the analyses included HIV plasma viral load, CD4 cell count, CDC clinical HIV stage, and time since antiretroviral therapy (ART) initiation at each follow-up visit. A detectable HIV viral load was defined as having a plasma HIV RNA level higher than the given hospital laboratory assay’s threshold. Information concerning diabetes and current ART status history was also available at each follow-up visit.

We used the body mass index (BMI) to classify patients as obese if the BMI was >30.

We also recorded information about HCV genotype, exposure to HCV treatment before enrolment and during follow-up, and post-treatment HCV clearance.

##### Variables in the Self-Administered Questionnaire

Data on patients’ socio-demographic characteristics (age, gender, educational level, marital status, and employment), coffee and tea consumption as well as psychoactive drug use were collected at enrolment and yearly thereafter using a self-administered questionnaire.

Data concerning patients’ tobacco use were recorded during face-to-face medical interviews with physicians. Patients were asked about their experience of smoking (non-smoker, former smoker, and current smoker).

The AUDIT-C questionnaire was used to assess alcohol consumption during the previous six months. The number of alcohol units (AU) consumed per day (a standard drink, defined as one AU, contains 11–14 g of alcohol, and corresponds to one small bottle of beer, one medium glass of wine, or a shot of distilled spirits) was calculated for patients who reported they were current consumers. Alcohol consumption was defined as “high-risk” if it was >4 AU/day for men and >3 AU/day for women, and “low-risk” if it was ≤4 AU/day for men and ≤3 AU/day for women [25]. Binge drinking was defined as reporting to have consumed six alcoholic drinks or more on one occasion.

Coffee intake was investigated using a question referring to the 6 months prior to the given follow-up visit. Five answers were possible: never, occasionally, 1 cup/day, 2 cups/day and 3 cups or more/day (1 cup corresponding to 150–200 mL). Patients were classified as having high coffee intake if they reported drinking 3 cups of coffee or more/day.

A four-category variable combining alcohol consumption and coffee intake was also created (low coffee intake and low-risk alcohol consumption, low coffee intake and high-risk alcohol consumption, high coffee intake and low-risk alcohol consumption and high coffee intake and high-risk alcohol consumption).

The self-administered questionnaire also collected information about psychoactive drug use consumption including use of cannabis and other drugs (cocaine, heroin, crack, ecstasy, street buprenorphine, amphetamines) in the month prior to the visit, as well as patients’ previous history of drug use.

### 2.3. Statistical Analysis

Participants’ characteristics at the last available follow-up visit with completed self-administered questionnaire were compared according to fibrosis status using a Chi-square test or Fisher’s exact test for categorical variables and Student’s *t* test for continuous variables. For continuous variables, means and standard deviations were calculated while for categorical variables we calculated proportions.

All the variables included in this statistical analyze were used as time-varying covariates, as these variables were collected at the baseline and at each follow-up visits, except for gender. We used mixed-effects logistic regression models in order to take into account the correlations between repeated measurements. This type of models enables testing of both fixed (e.g., gender) and time-varying covariates (e.g., consumption behaviors), In the univariate analysis, we identified explanatory variables correlated with fibrosis status. Those with a liberal *p*-value ≤ 0.25 were selected to be candidates for the final multivariable model.

The final multivariable model was built using a backward selection procedure, which was based on the likelihood ratio test (*p* < 0.05). Results were reported as adjusted odds ratios (aOR) with 95% confidence intervals (CI). Interactions between independent variables were also tested for.

Statistical analyses were performed using SAS software, version 9.4 (SAS Institute Inc., Cary, NC, USA).

## 3. Results

### 3.1. Patients’ Characteristics at the Last Available Follow-Up Visit with a Completed Self-Administered Questionnaire

A total of 1019 patients were included in the study with a median follow-up of 5.0 years (IQR: 4.1–6.0). Men accounted for 69.7%. At the last available follow-up visit with a completed self-administered questionnaire, one third of patients had at least a high-school certificate and almost half (48.2%) were employed. Patient age varied between 19 and 75 years with a mean (SD) age of 47.8 (±6.4) years. In addition, 15.2% had ALF. The majority of patients (95.0%) were receiving ART. Only 36.9% were receiving or had received anti-HCV treatment. Elevated coffee intake was reported by 27.4% of the study patients, and patients without ALF were more likely (*p* = 0.0002) to report elevated coffee intake (29.3%) than those with ALF (14.1%). Almost 6% of the patients reported high-risk alcohol consumption. Patients with ALF were more likely (*p* = 0.0018) to report high-risk alcohol consumption (11.3%) than those without ALF (4.7%) (Table 1).

### 3.2. Factors Associated with ALF

In the univariate analyses, the following variables were significantly associated with higher odds of having an ALF (*p* < 0.05): older age, unemployment, lower CD4^+^ cell count, obesity, not currently receiving ART, currently receiving HCV treatment, and high-risk alcohol consumption (Table 2). By contrast, high coffee intake and being HCV cured were significantly associated with lower odds of having ALF.

The multivariable analysis (Table 2) confirmed these results, except for the association with unemployment, which was no longer significant after multiple adjustment. Moreover, obesity increased the odds of having advanced fibrosis.

**Figure 1:**
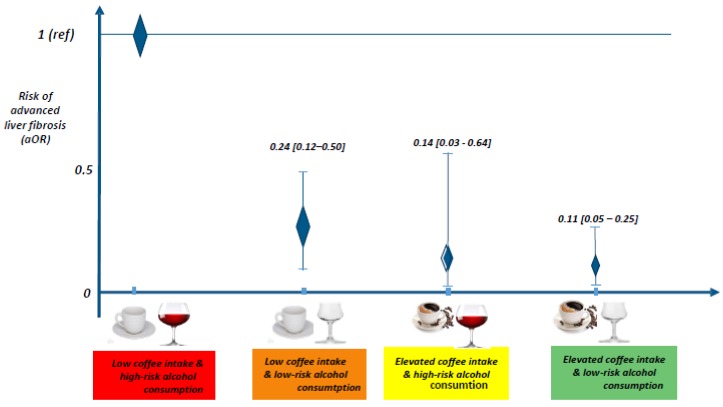
Risk of advanced liver fibrosis according to the pattern of coffee and alcohol consumption.

After multiple adjustment, compared with patients with low coffee intake and high-risk alcohol drinking who had a higher risk of advanced fibrosis (reference group), patients with low coffee intake and low-risk alcohol drinking had a 76% lower risk of ALF aOR (95% CI): 0.24 (0.12–0.50)). Among those with high coffee intake, high-risk alcohol consumption seemed to have no effect on liver fibrosis, with these drinkers having at least an 86% lower risk of advanced fibrosis than the reference group (0.14 (0.03–0.64) in high-risk alcohol drinkers and 0.11 (0.05–0.25) in low-risk alcohol drinkers) (Table 2 and Figure 1). 

## 4. Discussion

In this longitudinal observational study of HIV-HCV co-infected patients from the French ANRS CO13 HEPAVIH cohort, after controlling for age, CD4, HCV clearance, ARV treatment and BMI, we found that there is an inverse relationship between alcohol intake and coffee consumption on the risk of ALF. This is a major result in a population where liver disease may persist even after HCV clearance, because of HIV-related risk factors [26]. This study also confirms that there is a strong inverse association between high coffee consumption and ALF. This important finding provides further evidence of the beneficial effect of coffee consumption on clinical issues in HIV-HCV co-infected patients.

Our findings are consistent with those of Stroffolini et al. [18] in a study conducted in Italy among patients who had either chronic hepatitis B or C. Their study showed that the association between high-risk alcohol consumption and the risk of cirrhosis decreased in individuals who consumed at least 3 cups of coffee/day. It has also been demonstrated that coffee minimizes the harmful effect of high-risk alcohol consumption on the functioning of the body and consequently on the health of the individual [27,28]. In Japan, Oze et al. conducted a case-control study to analyze the association between coffee and tea consumption and the risk of upper aerodigestive tract (UADT) cancer [27]. They demonstrated that drinking three cups of coffee or more per day was inversely associated with incidence of UADT cancer, but that this protective effect was observed only among people who had never smoked or drunk alcohol. In addition, in a study on mortality among 28,561 individuals in a cohort from three Eastern European countries [28], a mortality study stratified on alcohol consumption showed that drinking three cups of coffee/day or more was inversely associated with mortality irrespective of the level of alcohol consumption.

Other studies have shown that even in patients with chronic liver diseases, coffee consumption was associated with a decreased risk of alcoholic related cirrhosis [29,30]

In prior studies conducted in the ANRS HEPAVIH CO-13 cohort, we showed that high coffee intake had the following beneficial effects in HIV-HCV co-infected patients: reduced levels of liver enzymes, including aspartate aminotransferase (AST), alanine aminotransferase (ALT) and gamma-glutamyltransferase (GGT) [15], fewer self-reported side-effects during peg-IFN and ribavirin treatment [20], and a 50% reduction in mortality risk [25]. These findings are consistent with those of a meta-analysis of studies on patients with chronic hepatitis C which also showed an inverse relationship between coffee intake and the risk of liver fibrosis [7,31,32].

Coffee is a complex mixture of bioactive components including caffeine and polyphenols, like chlorogenic acids, although the precise chemical ingredient profile depends on the variety of coffee. These substances not only decrease the inflammation of liver cells (in the case of liver disease) by reducing the expression of inflammatory cytokines, but also demonstrate a well-documented anti-fibrotic effect [33,34]. It is also well documented that high alcohol consumption is strongly associated with several hepatic complications, including hepatic inflammation, steatosis and ALF in HIV-HCV co-infected patients [35,36]. In these patients, the two diseases are independently involved in fibrinogenesis—the inflammation of hepatic cells—and hepatocyte apoptosis in the liver [37,38,39,40]. This predisposes these patients to a higher risk of developing liver fibrosis and cirrhosis. These mechanisms may be accelerated by high-risk alcohol consumption. However, in our study, the latter effect seems to have been greatly diminished in HIV-HCV co-infected patients who drank at least 3 cups of coffee/day. Although the protective effect of coffee for several major health issues is becoming increasingly plausible, the mechanism by which coffee intake slows the progression of liver disease in HIV-HCV co-infected patients, and/or how it may inhibit the toxic effect of alcohol on the liver, is not understood. Some studies have reported antioxidant properties of certain components of coffee such as chlorogenic acids [41,42]. These properties help regulate the genes involved in the fibrogenesis process, and this could partially explain why patients in our study who consumed coffee were less likely to have ALF. Furthermore, just as has been reported for the effect of certain nutritional supplements—including L-cysteine, vitamin C and vitamin B1—on alcohol toxicity [43], coffee might also interact in the metabolization of blood alcohol into acetate, carbon dioxide and water, and thereby minimize the toxic effect. Another explanation, is that high coffee consumption may be associated with decreased alcohol consumption or the blocking of specific alcohol receptors in liver cells. Future studies are needed to better understand the interactions between consumption behaviors, including alcohol and coffee intake, and liver-related outcomes, such as liver fibrosis and liver stiffness, in HIV-HCV co-infected patients.

As reported elsewhere [44,45,46], HCV clearance in HIV-HCV co-infected patients with sustained virological response (SVR) was associated with a lower probability of having ALF, meaning that HCV clearance after antiviral therapy had a major impact on the natural course of the disease. A previous study in this cohort showed that in HIV-HCV co-infected patients, SVR after pegylated interferon-based treatment was significantly associated with improvement in liver stiffness [46]. In another study conducted among HIV-HCV co-infected patients in Spain [45], those who were treated for chronic HCV and cured with peg-IFN and ribavirin, experienced a significant reduction in liver fibrosis. Among chronic HCV patients in the United States, Fontana et al. [47] showed that serum levels in fibrosis markers decreased significantly in patients with SVR after peg-interferon- and ribavirin-based treatment for 24 to 48 weeks. In addition, Berenguer et al. [48] evaluated the clinical course of a cohort of HIV-HCV co-infected patients who were followed even after therapy with peg-interferon plus ribavirin, and showed that patients with SVR had significantly fewer liver-related events, including liver fibrosis, than those without SVR. The primary goal of the peg-Interferon and ribavirin treatment is the eradication of HCV, which may slow, stop or even reverse the progression of HCV infection events including liver fibrosis. In addition, successful HCV treatment leads to hepatic inflammation reduction and liver function improvements, even in patients with decompensated cirrhosis or transplant patients with chronic hepatitis C. These beneficial results are now being amplified by the generalized use of Direct Antiviral Analogues (DAA), which enable the treatment and cure of a large majority of HIV-HCV coinfected patients. 

Importantly, our results revealed that co-infected patients on ARV treatment were less likely to have ALF. HIV infection is known to have a harmful effect on the natural history of HCV infection. In chronic hepatitis C patients, HIV co-infection was strongly associated with a rapid progression of hepatic complications including liver fibrosis and cirrhosis, due to immunosuppression [49,50]. Logically therefore, antiretroviral treatment in HIV-HCV co-infected patients should reduce the progression of liver complications [51]. However, several studies have shown persistent progression of liver fibrosis in HIV-HCV co-infected patients on antiretroviral treatment, which could be explained by a potential hepatotoxicity effect (including necroinflammation of hepatic cells) of certain categories of antiretroviral drugs [52,53]. In addition, closely related to the effectiveness of antiretroviral therapy, a greater CD4 count was associated with a decreased probability of having advanced fibrosis in the present work.

In our study, patients’ body mass index was significantly associated with the risk of liver fibrosis, as obese HIV-HCV co-infected patients were six times more likely to have ALF. Several studies have shown a strong association between obesity and disease progression in chronic HCV patients [54,55,56,57]. In a study of an American cohort of chronic HCV patients with available liver biopsies, Younossi et al. [57] highlighted that overweight and obese patients were much more likely to have advanced fibrosis. In another study of chronic HCV patients [56], obese patients had a greater risk of advanced fibrosis. Similar results were found by El-Ray et al. in Egypt [55]. The harmful effects of obesity are caused by a state of chronic metabolic inflammation induced in the liver, which may predispose individuals to liver fibrosis and non-alcoholic fatty liver disease. This result, in our study, should be interpreted with caution, as the confidence interval (5.93 (1.95–18.07), *p* = 0.0031) is wide.

Finally, results from a meta-analysis [58,59] suggest that the effect of coffee does not depend on caffeine, as similar benefits on liver diseases including hepatocellular carcinoma were shown for caffeinated and decaffeinated coffee.

Our study had several limitations. First, it was observational in nature, meaning that the associations observed did not imply causality. Accordingly, more research (for example a randomized clinical trial) is needed to confirm these findings in this population. Second, although the sensitivity of FIB-4 was estimated to be only approximately 65% in a different study by Sterling et al. [19]. The FIB-4 index is nonetheless considered one of the most reliable non-invasive methods in the assessment of liver fibrosis in HIV-HCV co-infected patients. In this study, we did not use data from a DAA-based cohort but from a PEG-IFN-based one, and so treatment initiation rates and cure rates were much lower. However, the positive effect of coffee on ALF remained true both for patients cured and not. Finally, the behavioral data related to the consumption of alcohol and other substances were based on self-reports which could be affected by social desirability bias.

## 5. Conclusions

This observational study analyzed the combined effect of coffee intake and alcohol consumption on the risk of ALF. High coffee intake was associated with a significantly reduced risk of ALF in HIV-HCV co-infected patients, even in those with high-risk alcohol consumption. This finding confirms the need to systematically take into account coffee intake in the evaluation of liver fibrosis progression in this population. Further studies are needed not only to confirm our findings, but also to evaluate the dose-effect response of coffee consumption on liver fibrosis in HIV-HCV co-infected patients.

## Figures and Tables

**Table 1:** Characteristics of HIV-HCV co-infected patients in the study population (*N* = 1019) at the last available follow-up visit with a completed self-administered questionnaire in the ANRS CO13 HEPAVIH cohort.

	*N* (%)	Advanced Liver Fibrosis ^1^		
		No, 822 (84.8%)	Yes, 147 (15.2%)	*p*-Value (chi-2)
Age, years				0.0856 *
Mean (±SD)	47.8 (±6.4)	47.4 (±6.3)	49.3 (±7.0)	
Median (IQR)	48 (44–51)			
Gender				0.2880
Male	710 (69.7)	568 (69.1)	108 (73.5)	
Female	309 (30.3)	254 (30.9)	39 (26.5)	
Living in a couple				0.8208
No	526 (51.9)	418 (51.0)	76 (52.1)	
Yes	488 (48.1)	401 (49.0)	70 (47.9)	
High-school certificate				0.1331
No	576 (66.4)	449 (65.2)	97 (71.8)	
Yes	291 (33.6)	240 (34.8)	38 (28.2)	
Employment				0.0100
No	525 (51.8)	406 (49.7)	90 (61.2)	
Yes	489 (48.2)	411 (50.3)	57 (38.8)	
CDC clinical stage				0.5139
Stage A	446 (45.1)	369 (45.0)	63 (42.9)	
Stage B	260 (26.3)	221 (26.9)	36 (24.5)	
Stage C	283 (28.6)	230 (28.1)	48 (32.6)	
CD4 count, cells/mm^3^				<0.0001 *
Mean (±SD)	564 (±309)	594 (±311)	394 (±355)	
Median (IQR)	516 (341–733)			
Body mass index ^2^				0.2155
Underweight or Normal	749 (78.4)	633 (79.6)	103 (74.6)	
Overweight	162 (17.0)	129 (16.2)	25 (18.1)	
Obese	44 (4.6)	33 (4.2)	10 (7.3)	
Diabetes				0.1888
No	1008 (98.9)	815 (99.1)	144 (98.0)	
Yes	11 (1.1)	7 (0.9)	3 (2.0)	
Receiving ART				0.5111
No	50 (5.0)	40 (4.9)	9 (6.3)	
Yes	951 (95.0)	770 (95.1)	135 (93.7)	
HCV treatment status				<0.0001
Not yet treated	643 (63.1)	518 (63.0)	98 (66.7)	
On treatment	62 (6.1)	36 (4.4)	19 (12.9)	
Treated, chronic HCV	108 (10.6)	79 (9.6)	25 (17.0)	
Treated, HCV-cured	206 (20.2)	189 (23.0)	5 (3.4)	
Alcohol consumption ^3^				0.0018
Low-risk	916 (94.2)	749 (95.3)	125 (88.7)	
High-risk	56 (5.8)	37 (4.7)	16 (11.3)	
Binge drinking ^4^				0.0699
No	725 (72.4)	594 (73.5)	96 (66.2)	
Yes	276 (27.6)	214 (26.5)	47 (33.8)	
Coffee intake				0.0002
Low	713 (72.6)	561 (70.7)	122 (85.9)	
High	269 (27.4)	232 (29.3)	20 (14.1)	
Cannabis consumption				0.5252
No	620 (71.8)	497 (71.4)	92 (74.2)	
Yes	244 (28.2)	199 (28.6)	32 (25.8)	
Tobacco consumption				0.4535
Never	116 (12.6)	102 (13.3)	13 (9.6)	
Past	147 (15.9)	123 (16.0)	21 (15.4)	
Current	659 (71.5)	543 (70.7)	102 (75.0)	
Coffee intake-alcohol consumption				<0.0001
Low coffee intake and high-risk alcohol consumption	39 (4.0)	25 (3.2)	12 (8.5)	
Low coffee intake and low-risk alcohol consumption	666 (68.9)	529 (67.8)	109 (77.3)	
High coffee intake and high-risk alcohol consumption	15 (1.6)	10 (1.3)	4 (2.8)	
High coffee intake and low-risk alcohol consumption	246 (25.5)	216 (27.7)	16 (11.4)	

* *t*-test; ^1^: FIB-4 ≥ 3.25; ^2^: Underweight or Normal (BMI < 25), Overweight (BMI between 25 and 30), Obese (BMI > 30); ^3^: High-risk alcohol consumption if >4 AU/day for men and >3 AU/day for women; and low-risk if ≤4 AU/day for men and ≤3 AU/day for women. ^4^: defined as reporting to have consumed six alcohol drinks (units) or more on one occasion during the previous 6 months.

**Table 2:** Factors associated with ALF in HIV-HCV co-infected patients, mixed logistic regression models, ANRS CO13 HEPAVIH cohort (*N* = 1019).

	Mixed-Effects Logistic Regression Models			
	Univariate Analyses		Multivariable Analysis	
	OR (95% CI)	*p*-Value	AOR (95% CI)	*p*-Value
Age, years	1.08 (1.05–1.1)	<0.001	1.18 (1.13–1.22)	<0.0001
Gender				
Male	1			
Female	0.67 (0.41–1.09)	0.109		
Living in a couple				
No	1			
Yes	0.91 (0.65–1.27)	0.559		
High-school certificate				
No	1			
Yes	0.66 (0.40–1.10)	0.114		
Employment				
No	1			
Yes	0.54 (0.40–0.74)	0.0002		
CDC clinical stage		0.463		
Stage A	1			
Stage B	1.25 (0.70–2.23)	0.417		
Stage C	1.37 (0.77–2.44)	0.252		
CD4 count ^1^, cells/mm^3^	0.85 (0.82–0.88)	<0.0001	0.74 (0.71–0.78)	<0.0001
Obesity ^2^		<0.0021		0.0031
No	1		1	
Yes	4.24 (1.78–10.10)	0.0021	5.93 (1.95–18.07)	0.0031
Diabetes				
No	1			
Yes	1.55 (0.75–3.21)	0.229		
Receiving ART				
No	1			
Yes	0.23 (0.13–0.39)	<0.0001	0.18 (0.09–0.34)	<0.0001
HCV treatment status		<0.0001		<0.0001
Not yet treated	1		1	
On treatment/Treated, chronic HCV	2.50 (1.79–3.49)	<0.0001	1.96 (1.33–2.90)	0.0008
Treated, HCV-cured	0.07 (0.04–0.13)	<0.0001	0.04 (0.02–0.08)	<0.0001
Alcohol consumption ^3^				
Low-risk	1			
High-risk	3.2 (1.8–5.7)	0.0002		
Binge drinking ^4^				
No	1			
Yes	0.85 (0.62–1.17)	0.3065		
Coffee intake				
Low	1			
High	0.49 (0.35–0.69)	<0.0001		
Cannabis consumption				
No	1			
Yes	1.09 (0.72–1.65)	0.687		
Tobacco consumption		0.6354		
Never	1			
Past	1.28 (0.28–5.93)	0.7402		
Current	1.67 (0.46–6.08)	0.4095		
Coffee intake-alcohol consumption		<0.0001		<0.0001
Low coffee intake and high-risk alcohol consumption	1		1	
Low coffee intake and low-risk alcohol consumption	0.24 (0.13–0.46)	<0.0001	0.24 (0.12–0.50)	0.0002
High coffee intake and high-risk alcohol consumption	0.26 (0.09–0.74)	0.0117	0.14 (0.03–0.64)	0.0114
High coffee intake and low-risk alcohol consumption	0.14 (0.07–0.27)	<0.0001	0.11 (0.05–0.25)	<0.0001

^1^: Intervals of 50 cell/mm^3^; ^2^: Underweight or Normal (BMI < 25), Overweight (BMI between 25 and 30), Obese (BMI > 30); ^3^: High-risk alcohol consumption if >4 AU/day for men and >3 AU/day for women; and low-risk if ≤4 AU/day for men and ≤3 AU/day for women; ^4^: defined as reporting to have consumed six alcohol drinks (units) or more on one occasion during the previous 6 months.
